# 950. Characteristics of Burn Patients Exceeding 50 Days' Length of Stay

**DOI:** 10.1093/jbcr/irag033.517

**Published:** 2026-04-06

**Authors:** Asha Cotterell, Alisa Savetamal, Elena Graetz, Eric B Schneider

**Affiliations:** Connecticut Burn Center, Yale-New Haven Health, Bridgeport, CT; Bridgeport Hospital/Yale New Haven Health, Bridgeport, CT; Yale University School of Medicine, New Haven, CT; Yale University School of Medicine, New Haven, CT

## Abstract

**Introduction:**

There are approximately 29 000 hospital admissions for burns annually in the United States. Length of stay (LOS) estimates range from 1 to 2 days per percentage total body surface area, but smaller burns, inhalation injury, or older age may exceed this calculation, and major burns (>20% TBSA) often have shorter LOS. At our center, we attempt to ensure that patients’ wounds are autografted or healed by 50 days due to the concern for mortality with delay in reepithelialization. This study was undertaken to identify the characteristics and outcomes of patients whose LOS exceeds 50 days to understand if this “50 day rule” remains a useful target.

**Methods:**

Adults admitted inpatient with a primary ICD-10 burn diagnosis were identified using the HCUP National Inpatient Sample (NIS) 2016-2021. Patients with a length of stay (LOS) greater than or equal to 50 days were identified. Differences in demographic and clinical characteristics as well as outcomes were assessed between patients with LOS < 50 days and LOS > = 50 days using chi-square tests and t-test.

**Results:**

A total of 146 455 patients were admitted inpatient with a primary burn diagnosis. Of these, 4065 (2.8%) had a LOS equal to or greater than 50 days. These patients (LOS > 50) were on average 48.9 years old, 68.7% were male, with no significant difference between LOS < 50 and LOS > 50 (all *p*>.05). LOS >50 patients had 3 or more comorbidities (61.6% vs 34% *p*<.01), higher mean rBaux score (88.6 vs 59.5, *p*<.01) and inhalation injury (13% vs 4.7%, *p*<.001). Patients with LOS >50 were more likely to undergo a skin graft (91.6% vs 48.9%, *p*<.01). 29.6% of burn patients with LOS >50 had acute kidney failure (vs 7.5%, *p*<.01) and were more likely to have acute respiratory failure (50.1% vs 3.4%, *p*<.01).

Of the patients whose LOS > 50 days, 9.6% died (vs 3.4% of LOS < 50, *p*>.05). Survivors were younger (48.2 vs 55.5, *p*<.01) with lower rBaux (86.7 vs 105.8, *p*<.01). There was no significant difference in number of comorbidities, inhalation injury, or number of skin grafts (*p*>.05 for all). Survivors had less acute respiratory failure (42.2% vs 76.9%, *p*<.001); acute kidney failure rates were similar (26.9% vs 29.9%, *p*=.58). Of patients discharged alive, 22% were routine discharges, 2.4% were transfers to short-term hospitals, 65.8% were other transfer (such as SNF), and 10.1% were discharged with home health care.

**Conclusions:**

A small minority of burn patients exceed 50 days' LOS. The survivors of this group tend to have smaller burns and lower Baux scores, and many (32%) are discharged with seemingly minimal needs for care, suggesting that other factors affected LOS. Further research into the timing of reepithelialization may give more concrete data to inform the validity of the 50 day rule.

**Applicability of Research to Practice:**

The mortality rate of patients in the LOS >50 group suggests opportunities for improving burn care with alacrity of operative management and with identifying patients appropriate for earlier discharge.

**Funding for the study:**

N/A.

